# Cleaning of in-hospital flexible endoscopes: Limitations and challenges

**DOI:** 10.1590/1518-8345.5969.3684

**Published:** 2022-10-17

**Authors:** Rosilaine Aparecida da Silva Madureira, Adriana Cristina de Oliveira

**Affiliations:** 1 Universidade Federal de Minas Gerais, Escola de Enfermagem, Belo Horizonte, MG, Brazil.

**Keywords:** Endoscopes, Gastrointestinal, Bacteria, Disinfection, Sterilization, Infection Control, Patient Safety, Endoscopios Gastrointestinales, Bacterias, Desinfección, Esterilización, Control de Infecciones, Seguridad del Paciente

## Abstract

**Objective:**

to analyze the cleaning process of gastroscopes, colonoscopes and duodenoscopes in eight in-hospital health services.

**Method:**

a cross-sectional study conducted with 22 endoscopes (eight gastroscopes, eight colonoscopes and six duodenoscopes), and microbiological analysis of 60 samples of air/water channels (all endoscopes) and elevator (duodenoscopes), in addition to protein testing. Descriptive statistics with calculation of frequencies and central tendency measures was used in data analysis.

**Results:**

the processing of 22 endoscopes was monitored with microbiological analysis for 60 channels. In the pre-cleaning procedure, in 82.3% (14/17) of the devices, gauze was used in cleaning the insertion tube. Incomplete immersion of the endoscope in detergent solution occurred in 72.3% (17/22) of the cases, and in 63.6% (14/22) there was no standardization of filling-in of the channels. Friction of the biopsy channel was not performed in 13.6% (3/22) of the devices. In the microbiological analysis, 25% (7/32) of the samples from the stored endoscopes were positive for microbial growth (from 2x10^1^ to 9.5x10^4^ CFU/mL), while after processing, contamination was 32% (9/28). Protein residues in the elevator channel were detected in 33% of duodenoscopes.

**Conclusion:**

the results indicate important gaps in the stages of pre-cleaning and cleaning of endoscopes that, associated with presence of protein residues and growth of microorganisms of epidemiological importance, indicate limitations in safety of the processing procedures, which can compromise the disinfection processes and, consequently, their safe use among patients subjected to such tests.

## Resumen

**Objetivo:**

analizar el proceso de limpieza de gastroscopios, colonoscopios y duodenoscopios en ocho servicios de salud intrahospitalarios.

**Método:**

estudio transversal con 22 endoscopios, de los cuales ocho eran gastroscopios, ocho colonoscopios y seis duodenoscopios, y análisis microbiológico de 60 muestras de los canales de aire/agua (todos los endoscopios) y elevador (duodenoscopios), además de prueba de proteínas. En el análisis de los datos se utilizó estadística descriptiva, con cálculo de frecuencias y medidas de tendencia central.

**Resultados:**

el procesamiento de los 22 endoscopios fue monitoreado con el análisis microbiológico de 60 canales. En la prelimpieza, en el 82,3% (14/17) de los equipos se utilizó gasa para limpiar el tubo de inserción. En el 72,3% (17/22) de los casos la inmersión del endoscopio en solución detergente fue incompleta y en el 63,6% (14/22) no hubo estandarización del llenado de los canales. La fricción del canal de biopsia no se realizó en el 13,6% (3/22) de los equipos. En el análisis microbiológico, el 25% (7/32) de las muestras endoscópicas almacenadas dio positivo para crecimiento microbiano (2x10^1^ a 9,5x10^4^ UFC/ml), mientras que después del procesamiento, la contaminación fue del 32% (9/28). Se detectaron residuos de proteína en el canal elevador en el 33% de los duodenoscopios.

**Conclusión:**

los resultados indican que hay importantes lagunas en las etapas de prelimpieza y limpieza de los endoscopios que, junto con la presencia de residuos de proteínas y del crecimiento de microorganismos de importancia epidemiológica, indican limitaciones en la seguridad del procesamiento, que pueden comprometer los procesos de desinfección y, por ende, el uso seguro en los pacientes que se someten a esos procedimientos.

**Highlights**

(1) No friction in the elevator channel.

(2) Friction of the channels with single diameter brushes in 63.6% of the endoscopes.

(3) Presence of protein in 33.3% of the samples analyzed.

(4) 25% of the samples from the stored endoscopes were positive for microbial growth.

(5) 32% of the samples from the endoscopes were contaminated, after processing.

## Introduction

The endoscopic equipment consists of a complex structure, long channels with extremely narrow lumens, which hinders the cleaning process, configuring a major challenge, taking into account the risk of the equipment remaining contaminated, if processing is not carried out properly and rigorously^1^.

Processing of an endoscope consists of numerous interdependent stages, namely: pre-cleaning, transportation, sealing test, cleaning, rinsing, disinfection, drying and storage^2^ ^-^ ^3^. For this process to be effective, it is necessary that all these stages are based on the implementation of best practices by the professionals working in Endoscopy services, meeting the evidence-based guidelines recommended by several societies and national/international bodies^1^ ^,^ ^3^. 

Rigor in complying with each of these stages is fundamental to control contamination of these devices after using them. Among all the stages, cleaning is considered a prerequisite of paramount importance for effective disinfection, as it aims at promoting removal of debris, blood and body fluids from the endoscope. Any deviation from the guidelines, recommendations and protocols can result in processing failure, culminating in infectious outbreaks, such as those recorded in the United States, France, Turkey and Spain and published between 2015 and 2020^4^ ^-^ ^7^.

Several publications that investigated dirt retention in the endoscopes’ channels indicate that cleaning can be one of the stages imposing the greatest challenge and an important bottleneck for safety in use of endoscopes; therefore, it is fundamental to implement tests that allow evaluating its effectiveness^8^ ^-^ ^9^.

In this context, it was sought to answer this question: Has the cleaning procedure for gastrointestinal endoscopes been performed effectively, providing safety for their processing?

Thus, the objective was to analyze the cleaning process of gastroscopes, colonoscopes and duodenoscopes in in-hospital health services.

## Method

### Study method, type, locus and population

This is an evaluative research study with a quantitative approach and a cross-sectional design, developed at in-hospital Gastrointestinal Endoscopy services specialized in upper digestive endoscopy, colonoscopy and duodenoscopy from Belo Horizonte, MG, Brazil.

The inclusion criteria indicated as potentially eligible services for the study those identified from the National Register of Health Establishments (*Cadastro Nacional de Estabelecimentos em Saúde*, CNES), with the premise that they were located in the hospital environment and performed upper digestive endoscopy, colonoscopy and duodenoscopy regardless of the number per month. Thus, eighteen services were identified. 

And, as an exclusion criterion, that hat had suspended activities during the data collection period, for reasons of health emergency caused by the serious COVID-19 pandemic period coinciding with the data collection period. Consequently, eight Endoscopy services were selected for the study.

In the services selected, processing was monitored and a microbiological analysis of 22 endoscopic devices was performed (eight gastroscopes, eight colonoscopes and six duodenoscopes), obtaining 60 samples from the air/water channels of all endoscopes and elevators (duodenoscopes).

### Study variables

The study variables were divided into five groups: factors related to characterization of the services (type of service administration; nature of the institution; type of processing adopted); pre-cleaning stages (external pre-cleaning of the endoscopes, as well as the device used; internal filling-in of the channels; type of cleaning solution used; professional category that performed the pre-cleaning stage) and cleaning stages for the equipment (immersion of the endoscope in the cleaning solution prior to brushing; type of detergent used; controlling immersion time of the endoscope in the cleaning solution; device used to inject the cleaning solution into the channels; standardization of detergent volume to fill the channels; controlling the temperature of the enzymatic detergent; and performing friction for all channels, as well as the type of brush used); and test used by the service to monitor the cleaning process.

In addition to that, conformity of the endoscope processing procedure was evaluated, being considered in compliance when absence of protein in the elevator channel after cleaning and absence of microorganisms in the microbiological culture from channel samples after processing (ready for use). 

### Period, instruments used for data collection, data collection

Data collection was conducted between February and June 2021, using a structured questionnaire prepared by the researchers and based on the recommendations of international societies on processing. All the questions allocated in the questionnaire were based on the Guideline published in consensus by the American Multisociety in 2021, which followed the classification of evidence described by the *Grading of Recommendations Assessment, Development and Evaluation* (GRADE), in addition to the documents prepared by renowned societies such as the European Society of Gastrointestinal Endoscopy and the European Society of Nurses and Gastroenterology Associates and the World Society of Gastroenterology. 

Application of the data collection instrument took place during observation of the processing of the endoscopes for issues related to pre-cleaning and cleaning, conducted by the main researcher who monitored this activity (mean duration of 40 minutes for each device). And its second part, related to the variables (in the case of reusable brushes, criteria established for disposal, standardized temperature for dilution of the enzymatic detergent, tests for monitoring the cleaning procedure), took place only through an interview without observation due to the type of information requested from the technician who performed the activity; at this time only needing to explain the practice for the processing of endoscopes in accordance with the recommendations of the service visited.

Five questions from the instrument referred to pre-cleaning and 24 to cleaning, including use of tests to verify this stage. The diverse information related to characterization of the service (three questions) was obtained by means of an interview with the person responsible for the service.

Data collection took place in a maximum of two visits, requiring the researcher to remain on site until completion of the exam schedule of that day. It is important to note that the process was monitored from pre-cleaning at the point of use to storage of the equipment. However, only the pre-cleaning and cleaning stages were addressed in this article. 

The technician invited to participate in the study was the one scheduled at the service for processing on the day of the visit. It is worth clarifying that this participation of the technician was voluntarily, after the invitation, guaranteeing him full autonomy in choosing to participate or not in the research, or even withdrawing his participation, without any kind of coercion, restraints or penalties. 

In order to evaluate effectiveness of the processing, samples were collected from the air/water channels (all endoscopes) and from the elevator (duodenoscopes) at two moments: stored equipment, before first use of the day; and after processing, at the end of the shift. These samples were subjected to microbiological analysis. In addition to that, protein testing was applied to the elevator channel after the cleaning stage. It is noted that the collection procedures were performed in the same devices at two moments, therefore sampling a total of 22 devices.

Sample collection from the air/water channels was carried out by the researcher, with the support of a Nursing undergraduate student duly trained for this activity. Aseptic technique was used, by means of the flush method, in which, with the aid of a syringe, 40 ml of double-distilled water were injected into the channel and the fluid obtained in the distal portion of the insertion tube was collected in a sterile container and sent for analysis^10^. For the elevator channel, the surface friction technique was used with the aid of a swab, described in other studies^11^ ^-^ ^12^. In this method, the elevator channel sample was obtained from all faces of the device (anterior and posterior), by friction with the aid of a swab and double-distilled water injection. 

After collection, the tubes with the samples were placed under refrigeration in a thermal box suitable for transportation and with temperature control, remaining between 2°C and 8°C, until they were sent to the laboratory, which was in charge of the researcher himself, in order to provide safety in sending the samples.

As for the method to perform the examination, it is noted that the culture was performed in an enrichment medium. Ten ml of the sample were used for enrichment and then one ml for each plate. The culture and isolation medium allowed for the growth of bacteria and fungi, specific to each group.

Bacterial identifications (except mycobacteria) were made through an automated method by MALDI-TOF mass spectrometry (Matrix-Assisted Laser Desorption/Ionization Time-of-Flight Mass Spectrometry - MALDI-TOF-MS). A VITEK-MS^®^ device from bioMérieux^®^ was used. Kirby-Bauer manual method (diffusion disk method) was adopted for the sensitivity tests, following the BrCast criteria. In the case of mycobacteria, identification varied on a case-by-case basis, by phenotypic methods, MALDI-TOF and molecular biology (PRA), being used alone or together.

The samples were processed in the national reference laboratory by the Brazilian Network of Analytical Laboratories in Health (*Rede Brasileira de Laboratórios Analíticos em Saúde*, REBLAS), authorized by the National Health Surveillance Agency (*Agência Nacional de Vigilância Sanitária*, ANVISA), a regulatory body linked to the Ministry of Health in Minas Gerais (Lacen-MG). 

### Data treatment and analysis

Data analysis was performed by means of descriptive statistics, with calculation of frequencies and central tendency measures and using the *Statistics and Data Science* (Stata) program, version 14.

### Ethical aspects

The current study was submitted to and approved by the Departmental Chamber of the Advisor and by the UFMG Ethics Committee under No. 4,574,663. Participation of the institutions, after consent, took place voluntarily and anonymously, without any financial benefit or coercion to participate. 

Participation of the professionals was formalized by signing the Free and Informed Consent Form.

## Results

Eight in-hospital Endoscopy services took part in the study, 75% of which were administered by the institution itself and 62.5% were private, i.e., they were services that provided care to individual patients with or without health insurance. Regarding the method used to process the endoscopes, the manual method was adopted in 50% of the devices and the mixed method (manual/automated), in the other 50%. 

The processing and microbiological analysis of 22 endoscopic devices (eight gastroscopes, eight colonoscopes and six duodenoscopes) were monitored, obtaining 60 samples from the air/water channels of all endoscopes and elevator (duodenoscopes). Although all endoscopic processing stages were observed, Table 1 presents and emphasizes those of pre-cleaning and cleaning because they were the main focus of this study.

**Table 1:** Analysis of the pre-cleaning and cleaning stages for the devices evaluated in the study (n=22). Belo Horizonte, MG, Brazil, 2021

Variable	Services (n) %
**Endoscope external pre-cleaning**	
Yes	77.3 (17)
No	22.7 (5)
**Internal filling-in of the endoscope channels during pre-cleaning**	
Yes	90.9 (20)
No	9.1 (2)
**Immersion of the endoscope in the cleaning solution for a time recommended by the manufacturer before brushing**	
Yes	22.7 (5)
No	72.3 (17)
**Device for injecting the cleaning solution into the channels**	
60 ml syringe	50.0 (11)
Pressure gun	36.4 (8)
Vacuum system	13.6 (3)
**Standardization of detergent volume to fill the channels**	
Yes	36.4 (8)
No	63.6 (14)
**Friction of all the accessible channels**	
Yes	86.4 (19)
Yes, except the biopsy channel	13.6 (3)

In relation to the external pre-cleaning of the endoscopes, it was verified that, among the devices subjected to this practice (n=17), gauze was used in 82.3% of the observations for the insertion tube. The compress soaked in the cleaning solution was used in 17.6% of the devices.

Internal pre-cleaning was performed in 90.9% (n=20) of the endoscopes and, of these, 95% were subjected to cleaning with enzymatic detergent and water was employed in 5%. This practice was in charge of physicians in 75% of the cases and of Nursing professionals in 25%.

Regarding cleaning, it was observed that 22.7% (5/22) of the devices underwent immersion in a detergent solution prior to brushing. In these cases, the endoscopes were kept completely immersed in the cleaning solution, using enzymatic detergent that, according to the institutions’ protocol, guided its dilution with water heated above 30°C. One service controlled temperature of the solution and was lower than recommended by the manufacturer, keeping it normally for use. In one institution, immersion of the endoscopes in the cleaning solution occurred after friction of the channels. The immersion time recommended by the manufactures for the devices was five minutes, which was respected by the services, as well as discarding the solution after each use. 

As for friction of the channels, it was verified that, in 63.6% (14/22) of the devices, all the channels were rubbed with single-sized brushes, without attention to differences in diameter between them. 

The processing of six duodenoscopes was observed, with friction of the elevator channel in 66.6% (4/6) of the devices. In a duodenoscope, articulation of the elevator mechanism was verified in order to access all its faces (anterior and posterior). For 50% of the duodenoscopes there were no brushes that were suitable/compatible with this channel.

Regarding monitoring of the cleaning procedure, 62.5% (5/8) of the services adopted this practice, with the adenosine triphosphate (ATP) bioluminescence test being standardized, with daily use frequency in 40% of the institutions, and weekly in 60%. The test was applied by sampling in the devices.

After the microbiological analysis, it was identified that, of the 28 samples obtained after processing and of the 32 from the stored equipment, 32% and 25%, respectively, were positive for growth of microorganisms (Figure 1).

**Figure 1:** Contamination frequency in the endoscopes after being processed and stored, according to the type of device and channel sampled (n=22). Belo Horizonte, MG, Brazil, 2021

Collection moment	Type of endoscope (channel sampled)	Samples collected (n=60)	Positive cultures (n=16)	Contamination rate (%)
After processing	Gastroscope (air/water)	8	4	25.0
	Colonoscope (air/water)	8	2	12.5
	Duodenoscope (air/water)	6	2	12.5
	Duodenoscope (elevator)	6	1	6.2
Stored	Gastrostoscope (air/water)	8	3	18.7
	Colonoscope (air/water)	8	3	18.7
	Duodenoscope (air/water)	8	1	6.2
	Duodenoscope (elevator)	8	0	0.0

All the endoscope models were contaminated. Regarding microbial load and collection moment, whether after processing or in stored equipment, Table 2 presents the findings according to the devices analyzed and the microorganisms identified.

**Table 2:** Microbiological analysis of the samples from the air/water/elevator channels of the endoscopes, according to service and collection moment (n=60). Belo Horizonte, MG, Brazil, 2021

Service ID*/ Type of device	Microorganism/ Resistance profile	Stored air/water channel (n=32)		Air/Water channel after processing (n=28)	
		Positive cultures (n=7) (%)	Microbial load (CFU^†^)	Positive cultures (n=9) (%)	Microbial load (CFU^†^)
SE^‡^ 1 Colonoscope	*Pseudomonas aeruginosa*, *Escherichia coli*, *Klebsiella pneumoniae* and *Kluyvera ascorbata* ^§^	14.3	>2.5x10^5‖^	¶	¶
SE^‡^ 3 Duodenoscope	*Mycobacterium tuberculosis* complex	¶	¶	11.1	<10
SE^‡^ 4 Gastroscope	Meropenem-resistant *Pseudomonas chlororaphis*	14.3	1.3x10^5^	¶	¶
	*Pseudomonas chlororaphis* ^§^	¶	¶	11.1	<10
	*Mycobacterium abcessus*	¶	¶	11.1	‖
SE^‡^ 4 Colonoscope	*Pseudomonas aeruginosa, Pseudomonas chlororaphis*; Meropenem-resistant	14.3	1.4x10^3‖^	¶	¶
SE^‡^ 5 Gastroscope	*Pseudomonas aeruginosa*, *Pseudomonas putida*, *Acinetobacter seifertii* ^§^	14.3	9.5x10^4‖^	¶	¶
	*Pseudomonas* sp^§^	¶	¶	11.1	<10
SE^‡^ 5 Colonoscope	*Stenotrophomonas maltophilia*; intermediate Sulfamethoxazole/trimethoprim	¶	¶	11.1	1.3x10^3‖^
SE^‡^ 5 Duodenoscope	Imipenem-resistant *Pseudomonas chlororaphis*	14.3	1.2x10^5^ ¶	¶	¶
SE^‡^ 5 Duodenoscope (elevator)	Chlororaphis Pseudonomas; intermediate to Imipenem	¶	¶	11.1	1.0x10^1^
SE^‡^ 7 Colonoscope	*Pseudomonas putida; intermediate to Imipenem; imipenem-resistant Serratia marcescens*	14.3	2x10^1‖^	¶	¶
SE^‡^ 8 Gastroscope	*Escherichia coli Pseudomonas aeruginosa, Serratia marcescens* ^§^	14.3	8.5x10^3‖^	¶	¶
	*Pseudomonas aeruginosa*; intermediate Imipenem	¶	¶	11.1	<10
SE^‡^ 8 Colonoscope	*Methylobacterium radiotolerans, Sphingomonas melonis*	¶	¶	11.1	3x10^1‖^

*ID = Identification; ^†^CFU = Colony Forming Unit; ^‡^SE = Service; ^§^Carbapenem-sensitive microorganisms; ^||^Sample with growth of more than one microorganism, with microbial load equivalent to the set; ^¶^No growth was detected

**Figure 2:**
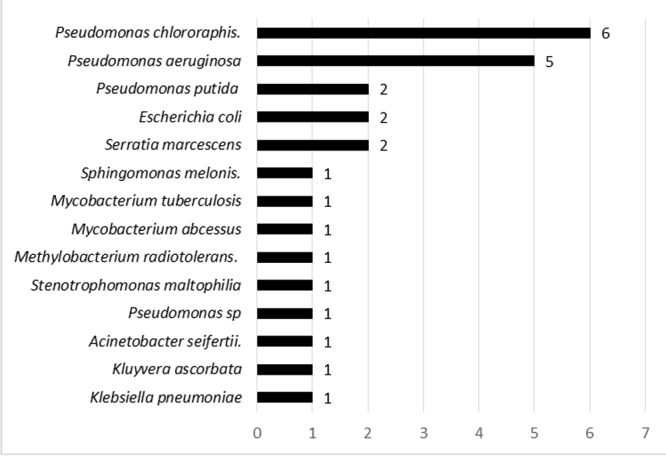
Distribution of the microorganisms identified in the air/water channels and elevator of the stored endoscopes or after processing (n=60). Belo Horizonte, MG, Brazil, 2021

The frequency of the microorganisms isolated in the devices, regardless of the sensitivity profile, is presented in Figure 2 below. 

In relation to the protein test, it was found that 33.3% (2/6) of the sampled duodenoscopes presented protein residues after cleaning, identified as devices whose elevator channel was not subjected to friction.

## Discussion

Although pre-cleaning of the endoscope channels at the point of use was performed on most of the devices 90.9% (20/22), external pre-cleaning drew the attention since, in 86.3% (19/22) of the endoscopes, this practice was not performed as recommended by the guidelines, that is, with the aid of a wet compress, soaked in detergent, throughout the length of the insertion tube. Use of dry gauze or gauze soaked in water was verified, which is not a practice recommended by the guidelines due to the risk of channel obstruction resulting from the lint, which can compromise integrity of the equipment, requiring its withdrawal from use and repair. In some cases, the obstruction can even cause exchange of equipment channel, reflecting in a high cost, in addition reduction of the technological supplies. 

On the other hand, exclusive use of water in pre-cleaning is not recommended, as it does not have the property of disaggregating organic matter inside the channels. The need to fill the channels with detergent is a way to meet the purpose of preventing drying of the secretions in the tube on its external surface, and especially inside the channels, in order to contribute to the cleaning process and avoid biofilm formation, disaggregating organic matter and other residues^1^ ^,^ ^13^ ^-^ ^16^. Therefore, detergent use as well as filling of the channels in adequate volume (injection of 200-250 ml) and their aspiration for a period of 10-20 seconds are fundamental in this stage^13^.

The importance of carrying out pre-cleaning of the endoscopes is widely highlighted among the recommendations by international societies^1^ ^,^ ^3^ ^,^ ^17^ ^-^ ^18^, and its inadequate execution, such as use of non-recommended inputs and solutions and non-standardization to fill the equipment channels, pointed out in this study, or its omission as recorded in another research study^19^, represent a considerable failure, which may compromise the safety of equipment processing.

After the pre-cleaning and sealing test, the recommendations by international societies suggest that the cleaning procedure itself should be initiated as soon as possible, between 30 and 60 minutes after the end of the examination. This stage comprises a set of interdependent actions, which include removal of all valves, irrigation of the channels and immersion of the equipment in detergent solution, followed by careful external and internal friction^1^ ^,^ ^13^.

In the current study, gaps involving immersion of the devices in enzymatic detergent were frequently found during the cleaning stage. It is noted that immersion of the equipment in the cleaning solution, prior to brushing and according to the time recommended by the manufacturer, did not occur in 72.7% (17/22) of the endoscopes analyzed. Omission of this step is an important flaw for efficiency of the cleaning procedure because, for the detergent to facilitate the reduction of dirt and microorganisms, it is considered essential that the endoscope and its accessories remain fully immersed for the time recommended by the manufacturer^1^ ^,^ ^3^ ^,^ ^15^.

Another important gap identified was friction of the channels with single diameter brushes in 63.6% (14/22) of the endoscopes observed. Adoption of this practice generates concerns regarding effectiveness of the cleaning procedure. The guidelines recommend using different brushes, with sizes and diameters compatible with each channel, so that the bristles have contact with the surface of the structures, in order to allow for the reduction of organic residues and microorganisms present in the equipment^14^ ^-^ ^15^ ^,^ ^17^. In order to avoid cross-contamination across the devices, the brushes should preferably be single-use^13^. If it is not possible, when reusable, it is indispensable that, after each use, they are subjected to high-level cleaning and disinfection or sterilization^13^ ^,^ ^20^.

When it comes to duodenoscopes, especially the models consisting of an elevator channel with fixed protection, the challenge for cleaning is even greater because the device does not allow reaching all its faces by brushing, with the part located after the elevator mechanism being the most difficult to access^21^.

Also contrary to the scientific recommendations, among the six duodenoscopes evaluated, in only one device the elevator channel was properly rubbed, that is, promoting articulation of the elevator mechanism and with a brush compatible with the channel. In the institutions where the automated method was adopted, most of the professionals were unaware that the elevator mechanism should remain upright throughout the process, in order to allow greater contact with the cleaning and disinfection solutions^3^ ^,^ ^14^. The inadequate position of this device deserves to be highlighted due to its potential accumulation of dirt and microorganisms, especially on its posterior face, which may favor maintenance of microorganisms in the structure^22^.

Such findings give rise to a special look at this stage of the process, as the professionals’ non-perception of the elevator mechanism as a threat to safe use of the duodenoscopes is a matter of concern^23^, as failures during its processing have been attributed as important causes of several infectious outbreaks, with involvement of numerous patients in different countries in the world^16^ ^,^ ^24^ ^-^ ^25^. This lack of knowledge reinforces the need to train Endoscopy teams more frequently on correct cleaning and disinfection of these devices^22^ ^-^ ^23^.

Given the major challenge for the effectiveness of endoscope cleaning, a number of societies have emphasized the importance of implementing methods that enable evaluation of this process^3^ ^,^ ^13^. The current study identified that 62.5% (5/8) of the services adopted tests for validation of the cleaning procedure in their routine. In these institutions, the ATP bioluminescence test was used as a potential marker of cleaning adequacy, with up to 200 Relative Light Units (RLUs) being considered as an acceptable value^26^. However, it is important to mention that the result of this test should be read with caution, as there is variability in the scale of the reference values according to each manufacturer. Thus, it is fundamental that the brand of the product used and its respective guidelines are observed, in order to avoid misinterpretations in the results^27^. 

It is also worth noting that ATP has been indicated as an important tool to monitor the cleaning technique performed by the professionals^8^ ^,^ ^28^ ^-^ ^29^. However, ATP detection after cleaning a health product represents uptake of live cell energy. Thus, its use needs to be careful since, although ATP provides values that meet the references, there is the possibility of the presence of non-viable cells in the channels, which may compromise effectiveness of the processing procedure and which will certainly not be detected by this test. For this reason, although useful as a marker for the cleaning process, this technology is not sensitive enough to be used as a marker for absence of microorganisms, and this analysis should be performed exclusively through microbiological cultures^30^. 

Considering these limitations for the ATP test, to verify cleaning of the equipment, we opted in this study for the use of the protein test, as this residue can act as an important substrate for biofilm formation in the endoscope channels. Thus, in the duodenoscope elevator channel, protein was found to be present after cleaning in 33.3% (2/6) of the devices evaluated. The result clearly portrays non-compliance with the scientific recommendations, as these channels were not rubbed during cleaning of the devices. In another study^8^, the authors drew the attention to the fact that, if protein is detected after manual cleaning, there is a probability that this residue will also be detected after high-level disinfection, which may imply toxic reactions to the patient and inefficient disinfection/sterilization, in addition to an increased risk of biofilm development and potential pathogen transmission^31^.

In other studies^32^ ^-^ ^33^, positive tests of protein applied in the endoscope channels also had an important relationship with non-adherence to the recommendations in the cleaning stage. In an experimental study it was clearly shown that absence of friction of the channels during cleaning can directly imply maintenance of microorganisms in these structures after processing, which can impair safe use of the equipment^33^. 

In relation to the microbiological analysis, microbial growth was detected in 45.4% (10/22) of the devices analyzed. Microorganisms indicating failures in processing were recovered, such as the following: *Serratia marcescens, Escherichia coli, Pseudomonas putida, Pseudomonas aeruginosa, Pseudomonas chlororaphis, Klebsiella pneumoniae, Acinetobacter seifertii, Pseudomonas* sp*, Stenotrophomonas maltophilia* and mycobacteria.

As for the resistance profile, of the 60 samples obtained from the equipment channels, 16 were positive and, of these, 28.5% corresponded to carbapenem-resistant *Pseudomonas* species and 21.4% presented an intermediate profile (sensitive, increased exposure), thus arousing even more concerns, considering the countless records of infectious outbreaks caused especially by *Pseudomonas aeruginosa* in several countries of the world, possibly due to processing failures^5^ ^,^ ^7^ ^,^ ^34^.

Recovery of mycobacteria species (*Mycobacterium tuberculosis* and *Mycobacterium abcessus*) is also noted. Outbreaks involving mycobacteria and patients subjected to endoscopic procedures have been recorded, particularly after bronchoscopy procedures^35^ ^-^ ^37^. The authors pay attention not only to inadequacies in the processing of endoscopes, surveillance of internal damage in the equipment and quality of the rinse water for the equipment, but also to the frequency of filter exchanges in the automated reprocessors, potential sources of contamination of endoscopes by mycobacteria^36^ ^-^ ^37^.

It should also be noted that fast-growing mycobacteria species caused a major outbreak in Brazil between 2003 and 2009, with more than two thousand cases reported from the North to the South of the country. The outbreak was strongly associated with surgical procedures and diagnoses by videoscopy, whose surgical instruments were subjected to sterilization through Glutaraldehyde^38^.

The pathogens identified in this study as *Escherichia coli*, *Pseudomonas aeruginosa* and *Mycobacterium* spp., regardless of the microbial load, are indicators of failures in the cleaning/disinfection process of the devices and can serve as a warning for Endoscopy services and, especially, for the professionals involved in processing of the endoscopes. Such microorganisms are not acceptable in ready-to-use equipment, especially due to the potential for cross-transmission, which can cause serious infections in patients subjected to endoscopic procedures.

Thus, when detected, it is fundamental that those responsible pay attention to a detailed review of the entire process, which implies precautions from pre-cleaning to handling the equipment after storage.

The findings of this research allowed outlining an overview of how the services have processed the endoscopes and met the recommendations set forth in the national and international guidelines. Practices that presented weaknesses and limitations, not meeting the scientific evidence, were identified in all participating services, and may be used as an indication for the policies regarding training of the teams working in Endoscopy services.

Regarding the study limitations, it is noted that the professionals’ knowledge about this research during monitoring of the processing of endoscopes may have favored occurrence of the “Hawthorne effect”, overestimating the quality of the practices observed. Consequently, given the identification of countless gaps in the process, it can be inferred that they can be even greater in the routine of the services.

In addition to that, despite having evaluated an important number of equipment channels (n=60) and having had the opportunity to observe multiple processing of 22 devices, allowing monitoring various process practices, the scenario experienced because of the COVID-19 pandemic definitely impacted on compliance with the schedule of the activities proposed and implied a lower than expected number of services. This impact was reflected in the refusal of six services considering that, due to the pandemic, field research studies, although previously accepted, were suspended in those places. In addition, a service that was undergoing a total change of management precluded conduction of the research. Therefore, the data were collected in eight services, which, despite the setbacks, still represented an important number of services monitored.

## Conclusion

The results of this study point to important gaps in the stages of pre-cleaning and cleaning of endoscopes that, associated with presence of protein residues after cleaning and growth of microorganisms of epidemiological importance, indicate limitations in safety of the processing procedures, which may compromise the disinfection processes of such devices and, consequently, their safe use among patients subjected to such tests.
